# TCM monotherapy achieves significant efficacy in crizotinib-refractory advanced NSCLC with brain metastasis

**DOI:** 10.1097/MD.0000000000034138

**Published:** 2023-07-21

**Authors:** Jiqiu Qin, Ru Li, Hong Ma, Peng Ding, Qin Yang, Lilai Hu, Deliu Wu, Shaoquan Xiong

**Affiliations:** a Department of Medical Oncology, Hospital of Chengdu University of Traditional Chinese Medicine, Chengdu, China.

**Keywords:** ALK rearrangement, brain metastasis, non-small cell lung cancer, traditional Chinese medicine

## Abstract

The morbidity and mortality of lung cancer rank the first among all kinds of cancer. In China, anaplastic lymphoma kinase-positive pulmonary tumors account for nearly 5% of non-small cell lung cancer (NSCLC), and these patients are quite likely to develop brain metastases, as high as around 45%. Although anaplastic lymphoma kinase-tyrosine kinase inhibitors crizotinib and alectinib have proved effective for controlling tumor metastases to the brain, drug resistance and disease progression cannot be ignored in the course of treatment.

**Patient concerns:**

Most of the literature reports that traditional Chinese medicine (TCM) has produced satisfactory results in the treatment of cancer patients as an adjuvant treatment for various malignancies in a 53-year-old male patient who developed advanced NSCLC with brain metastases. As first-line crizotinib and erlotinib treatments were ineffective and the intracranial lesions progressed extensively, the patient chose to receive TCM treatment alone in the hope of prolonging his life and improving his quality of life.

**Diagnoses:**

A 53-year-old male patient who developed advanced NSCLC with brain metastasis. Because first-line crizotinib and alectinib have failed, and the intracranial lesions progressed in a large area.

**Interventions:**

The patient requested that the final therapeutic strategy be Chinese medicine as monotherapy for long-term treatment. The patient took 30 mL of the decoction 1 hour after a meal, 3 times a day. The patient was not treated with dehydrating agents or diuretics during the TCM treatment.

**Outcomes:**

The improvement was obvious after 3 months of treatment, and significant reduction of cranial lesions. During the follow-up period, the patient developed neither severe liver damage nor kidney damage.

**Lessons:**

This case is the first 1 in the world where TCM was introduced as monotherapy for severe conditions with extensive brain metastases and achieved remarkable efficacy.

## 1. Introduction

Lung cancer ranks first in the morbidity and mortality of all kinds of malignant tumors. According to the latest authoritative report in China, lung cancer was the first morbidity of cancer in males and the second highest in females, which was reported as a major cause of cancer death in 2015. Non-small cell lung cancer (NSCLC) consists of around 85% of all lung cancers, with only a 16% of 5-year survival rate for most patients.^[1]^ anaplastic lymphoma kinase (ALK) gene rearrangements are the fundamental mutation of NSCLC, which are found in 5% to 6% of NSCLC cases.^[2]^ ALK-tyrosine kinase inhibitors (TKIs), both the first and second generations, have been introduced as the standard therapy for patients with ALK-positive NSCLC.^[3–6]^ The first-line standard crizotinib has been recommended by NCCN guidelines for untreated advanced NSCLC patients with ALK rearrangement. Their median progression-free survival (PFS) has been reported to be 10.2 to 11.1 months and the objective response rate is 59.8% to 74%.^[3,5,7,8]^

However, most NSCLC cases progress, predominantly the central nervous system (CNS) 1 year after receiving the first-line crizotinib treatment. Among the NSCLC patients, 40% of them have developed brain metastases (BM),^[9]^ and it has been reported that BM occurs in around 30% of patients on diagnosis.^[10]^ Unfortunately, they are reported to have a depressing prognosis with a median survival of fewer than 5 months.^[11]^ Untreated patients with brain metastases have a median overall survival (OS) of only 1-2 months,^[12]^ so repeated intervention for BM is very urgent. The consensus of experts in China in diagnosing and treating BM of lung cancer suggests asymptomatic BM patients should be initiated systemic treatment (ALK-positive patients receive first-line crizotinib), and patients with symptomatic BM and stable extracranial lesions should be actively treated locally. Conventional treatment options for this disease are surgical excision, stereotactic radiosurgery (SRS), whole brain radiotherapy (WBRT), chemotherapy, targeted therapy, or combined therapies to treat metastatic lesions, relieve symptoms, prolong survival time as well as improve the quality of life, but these efficacies are disappointing.

In recent years, traditional Chinese medicine (TCM) has been broadly applied in the treatment of malignant tumors, which has shown excellent results in improving the efficacy of chemotherapy and radiotherapy and minimizing the toxicity of both therapy options. However, at present, there is still a lack of an effective treatment for the NSCLC progress in CNS with the unassured survival status of such patients. We presented the typical case of TCM monotherapy achieving remarkable efficacy in the treatment of ALK-positive, crizotinib resistance, and refractory advanced NSCLC with BM, and focused on the feasibility of TCM to alter the prognostic potential of this disease.

## 2. Case data

**Figure 1.**
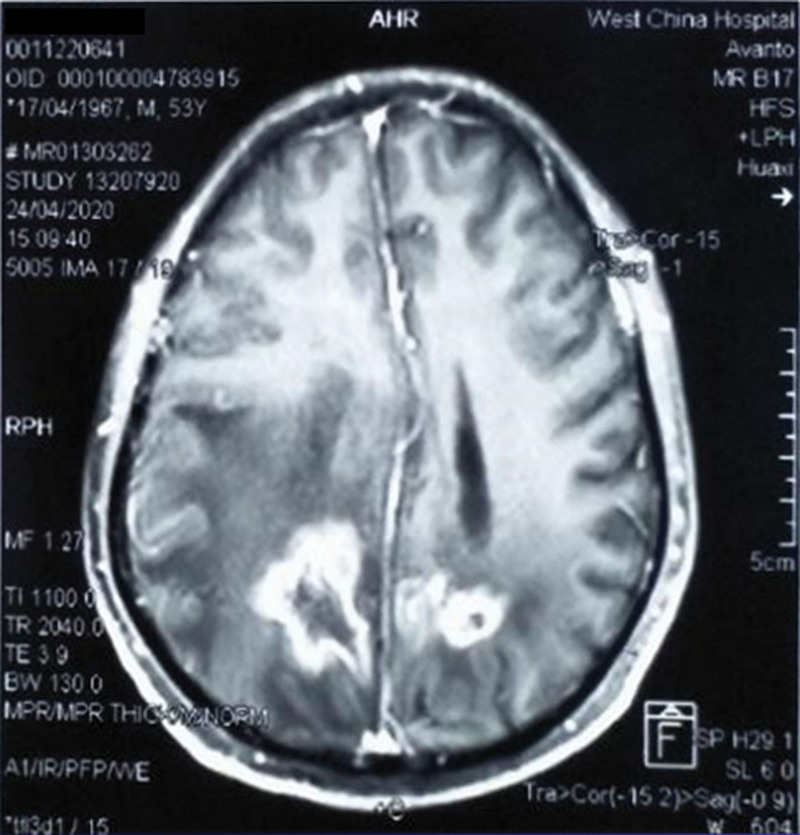
In April 2020, MRI indicated the larger section of a target lesion in the parietal lobe at 5.1 × 4.1 cm in size. MRI = magnetic resonance imaging.

**Figure 2.**
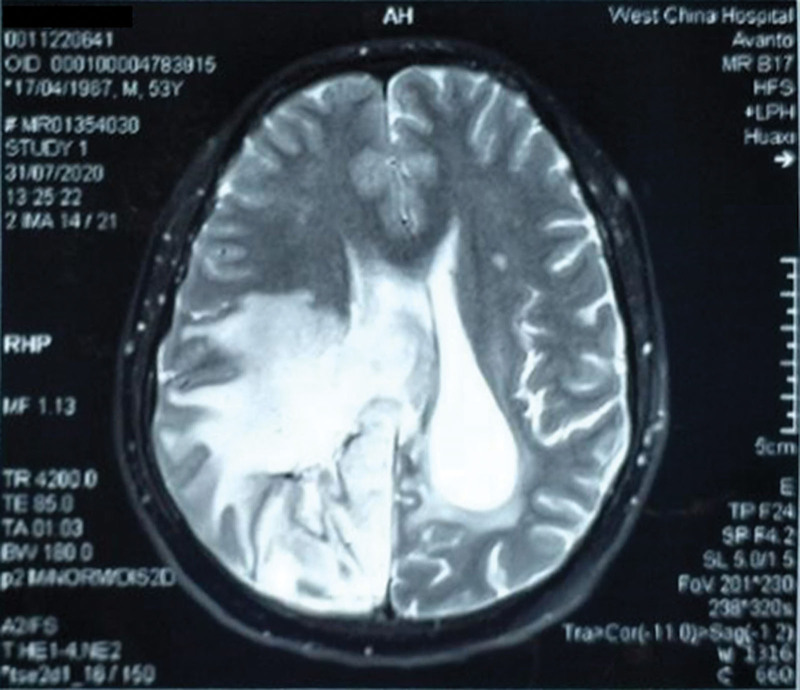
In July 2020, MRI indicated the size of the target lesion at 5.3 × 4.6 cm. MRI = magnetic resonance imaging.

A 53-year-old male with stage IIIa adenocarcinoma in the right lung underwent surgery in 2014 and BM was observed in the head in 2018. The rearrangement of the ALK gene was confirmed by postoperative gene detection. In September 2018, due to dizziness and headache, a brain magnetic resonance imaging (MRI) scan showed that the larger lesion was in the right parietal lobe with a long diameter of about 4.5 cm. The left ventricle was deformed under compression, and the center line deviated to the right. Then crizotinib was treated for 18 months. In April 2020, the patient experienced suddenly aggravated dizziness and headache, limb weakness, and difficulty walking. The MRI examination results indicated a target lesion in the parietal lobe at the size of 5.1 × 4.1 cm, with obvious edema in the surrounding cerebral parenchyma (Fig. 1). Alectinib was used instead. In July, at baseline, reexamined MRI scan revealed the target lesion of the larger section in the parietal lobe was 5.3 × 4.6 cm in size (Fig. 2).

After informing the patient and his family about their condition and standard treatment measures, they were afraid of the side effects of radiotherapy and chemotherapy. As a result, they requested that the final therapeutic strategy be Chinese medicine as monotherapy for long-term treatment. The components of the prescription were as follows: *Radix Rehmanniae Praeparata* 15 g, *Rhizoma Dioscorea* 15 g, *Rhizoma Arisaematis* 9 g, *Bombyx Batryticatus* 15 g, *Coptis Chinensis* 6 g, *Fermentum Rubrum* 12 g, *Semen Sinapis* 10 g, *Fructus Lycii* 15 g, *Cyathula Officinalis* 15 g, *Achyranthes Bidentata* 15 g, *Gastrodiiae Elata* 20 g, *Astragalus Membranaceus* 100 g, *Phellodenron Amurense* 20 g, *Cornus Officinalis* 30 g, *Ziziphus Jujuba* 15 g, *Fructus Ligustri Lucidi* 15 g, *Acorus Tatarinowii* 15 g, *Polygala Tenuifolia* 15 g, and *Eclipta Prostrata* 15 g. These herbs were added into 500 mL water, boiled with high fire, and continued to decoct for about 1 hour with low fire. Then the decoction was concentrated to 90 mL. The patient took 30 mL of the decoction 1 hour after a meal, 3 times a day. The patient was not treated with dehydrating agents or diuretics during the TCM treatment.

**Figure 3.**
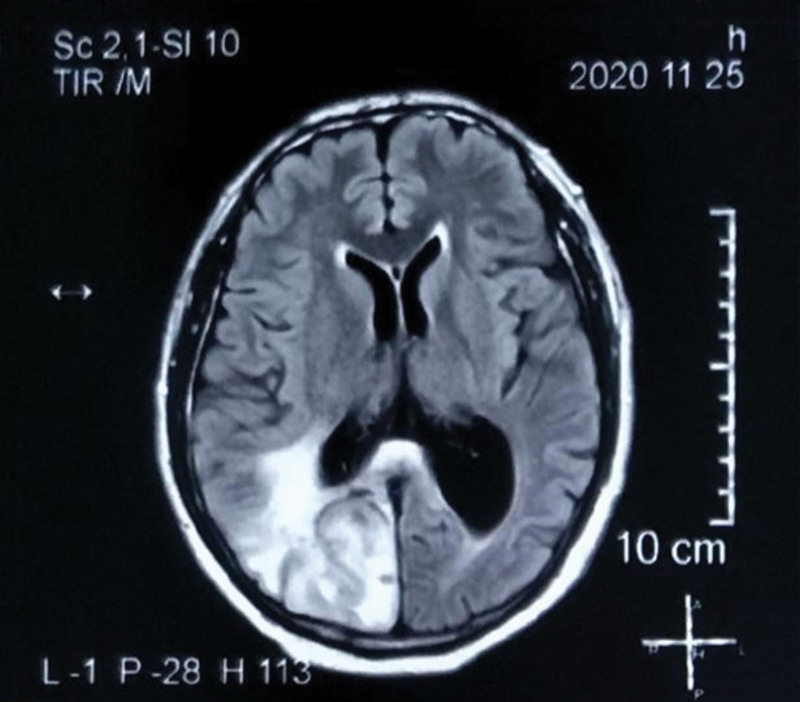
In November 2020, MRI indicated a smaller size of 2.7 × 1.2 cm of the lesion in the right occipital lobe. MRI = magnetic resonance imaging.

**Figure 4.**
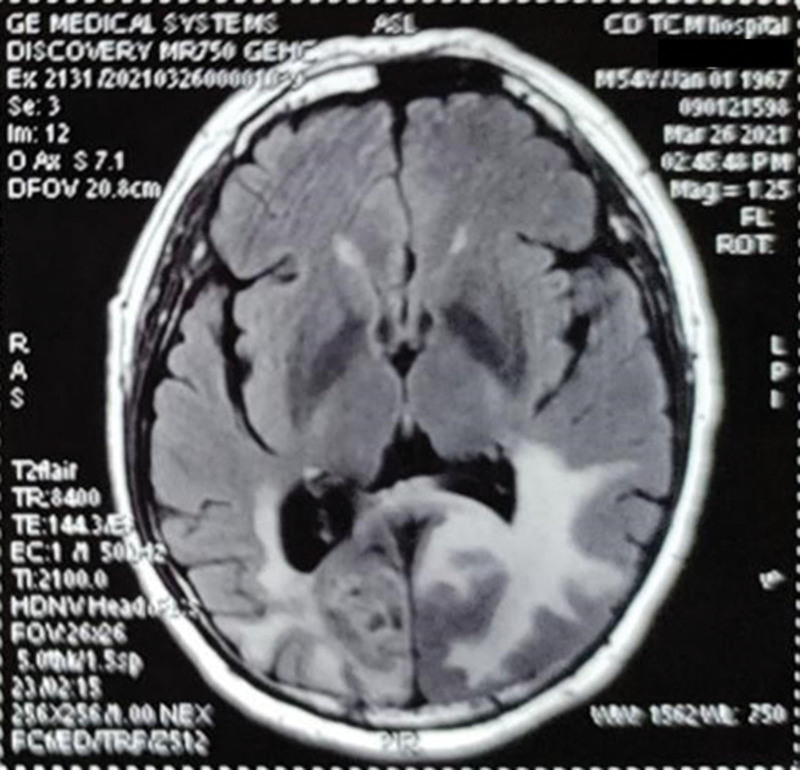
In March 2021, MRI indicated a smaller size at 3.2 × 3.4 × 4.5 cm of the lesion. MRI = magnetic resonance imaging.

The improvement was obvious after 3 months of treatment. The patient resumed walking upright for more than half an hour every day, and the symptoms of dizziness, headache, cough, expectoration, poor appetite, and loose stools were significantly relieved. In November, the patient accepted MRI scanning again and the lesion in the right occipital lobe was reduced to 2.7 × 1.2 cm (Fig. 3). Compared with the baseline, it was significantly narrowed, reaching partial remission. In March 2021, the patient took the decoction for more than 6 months. He could walk as freely as a healthy individual and perform general physical work, and dizziness symptoms basically disappeared. The target lesion was 3.2 × 3.4 × 4.5 cm according to the MRI examination (Fig. 4). During the follow-up period, the patient developed neither severe liver damage nor kidney damage.

## 3. Discussion

The alectinib objective response rate in the central nervous system was 54.2% in ALK-positive brain metastases patients who had previously received first-line therapy progression.^[13]^ In this report, the patient did not achieve satisfactory results during the 3 months of treatment with alectinib. Due to the progression of the intracranial lesions and the worsening of the symptoms, the patient came to our hospital seeking treatment. Standard therapies including single-agent chemotherapy,^[14]^ immunotherapy,^[15]^ and WBRT^[16]^ seldom produce satisfactory or better effects in the second-line setting. The median PFS for a patient to accept the chemotherapy option is 1.6 months, and it is introduced for the treatment of advanced/metastatic ALK-positive NSCLC victims who have selected treatment strategies of doublet chemotherapy and crizotinib based on platinum, and it was ineffective for intracranial metastasis.^[13]^ ALK-positive patients present poorly expressed PD-L1 as compared to those with negative ALK, and the PFS lasted only 1.2 to 2.1 months.^[15]^ For localized brain metastases (brain metastases < 4 cm), SRS plus whole brain radiotherapy can reduce the recurrence rate of the CNS, but cannot improve the overall survival rate, with a median OS of only 5.8 months^[5]^ and an increased risk of cognitive decline. WBRT is regarded as a standard treatment for NSCLC patients with multiple brain metastases, with a response rate of approximately 40% to this therapy option.^[16]^ Unfortunately, over 50 percent of them treated with WBRT could not manage to survive due to progressive systemic disease.^[17]^ In a study of concurrent erlotinib plus WBRT for patients with multiple brain metastases, only 1.6 months and 3.4 months were achieved for the median neurological PFS and OS.^[11]^ The newly developed third-generation ALK-TKI lorlatinib has shown superior efficacy over its predecessors in simple cases but has not yet shown significant efficacy in the complex cases described in this report. A study has revealed that the median PFS of BM patients was 6.9 months who had previously been administered with ≥ 2 ALK-TKIs with or without chemotherapy.^[18]^ Combined with the effect of existing treatment measures and the patient’s wishes, we finally chose to use TCM alone to treat this patient.

TCM boasts a long treatment history over the past 2000 years as a widely accepted strategy against various diseases in China. In recent years, as a kind of adjuvant and alternative therapy, Chinese medicine is attracting increasing attention, and it has shown remarkable effects in improving curative effects and alleviating adverse reactions. At present, it is believed that TCM, as a complementary therapy for cancer, is of vital importance during the entire process of cancer treatment (including postoperative recovery, radiotherapy, and chemotherapy).^[19]^ In a survey on TCM application in cancer patients from 4 southern Chinese provinces, 54.61% of patients were willing to initiate treatment by applying TCM and 14.46% chose TCM as monotherapy. It was believed by 81.08% of patients who received 1-month TCM treatment that taking TCM before anticancer treatment was effective.^[20]^ These showed that Chinese medicine has achieved amazing results in these patients. This report also pointed out that tumor growth has been controlled in 8.31% of patients with declined tumor markers, indicating that TCM revealed precise anticancer effects in clinical practice, which is worthy of further exploration and research and may provide a new approach for the treatment of malignancies. The median OS of NSCLC patients at stages III to IV after TCM treatment was higher than standard therapy (16.60 m vs 13.13 m).^[21]^ In addition to the significant effect of TCM on lung cancer, the median OS of pancreatic cancer patients who accepted TCM treatment (15.2 m) was better than combination chemotherapy of FOLFIRINOX (11.1 m).^[22,23]^ In the above-mentioned reports, only a few patients reported that they experienced side effects including nausea, vomiting, diarrhea, and inappetence during TCM treatment.^[20,21]^ These studies have proved the admirable safety of Chinese medicine.

In recent years, TCM, as adjuvant and alternative therapy, extensively used in cancer treatment (including recovery stages of postoperative, radiotherapy, and chemotherapy). Many benefits have been achieved in controlling tumor progression, alleviating postoperative complications, improving sensitivity to chemotherapy and radiotherapy, enhancing immune system functions, as well as relieving relevant damages associated with surgeries, chemotherapy, and radiotherapy.^[19]^ The international mainstream view of TCM is used for the palliative treatment of terminal cancer nowadays.^[24,25]^ However, in this report, a middle-aged man was diagnosed with advanced NSCLC with BM, and first-line treatment with crizotinib and aletinib failed and the disease continued to progress. According to NCCN guidelines, patients were advised to choose WBRT or SRS, but this patient refused radiotherapy because of fear of its adverse reactions. Therefore, when radiotherapy, targeted therapy, and surgery are not available, TCM therapeutics are our only means of treatment. After 3 months of treatment, reexamined MRI scanning indicated a reduction of target lesion size by 2.6 cm (5.3 cm vs 2.7 cm). In March 2021 (after 9 months of TCM treatment), MRI showed that the intracranial lesions had increased, but they were still smaller than the size at the initial diagnosis. The patient’s symptoms had basically disappeared, and he was living independently. By the time of writing this report, the patient has been able to walk normally and live completely individually. As far as we know, Chinese medicine, as the only treatment for advanced NSCLC with BM, has shown surprising efficacy in a short period, which is the first successful case in the world. During the follow-up, no damage to the liver or kidney was revealed. Therefore, is it reasonable to speculate that TCM can achieve better OS in advanced BM patients, which is generally safe and easily tolerated?.

Experiments proved that Chinese herbal medicine and its extracts can inhibit the growth of tumor cells or prevent the occurrence and development of tumors in various ways. TCM, such as *Rhizoma Dioscorea*,^[26]^ *Rhizoma Arisaematis*,^[27]^ *Bombyx Batryticatus*,^[28]^ *Coptis Chinensis*,^[29]^ *Fructus Lycii*,^[30,31]^ *Cyathula Officinalis*,^[32]^ *Astragalus Membranaceus*,^[33–35]^ *Phellodenron Amurense*,^[36]^ *Cornus officinalis*,^[37]^ *Gastrodiiae Elata*,^[38]^ *Ziziphus Jujuba*,^[39]^ *Polygala Tenuifolia*,^[40]^ and *Eclipta Prostrata*^[41]^ have been confirmed the effect of antitumor. Berberine, the extract of *Rhizoma Coptidis*, can directly induce apoptosis.^[42]^ *Ziziphus Jujuba* can delay the progression of colon cancer from hyperplasia to dysplasia and eventually adenocarcinoma and cancer,^[39]^ and Astragalus membranaceus combined with Angelica sinensis can inhibit lung cancer cell growth through immunomodulatory function.^[34]^ However, due to the replicability of decoction components, the antitumor mechanism of TCM is still unclear and needs further study.

## 4. Conclusion

This report shows that TCM can acquire satisfactory results in ALK-positive, crizotinib-refractory advanced NSCLC with BM. TCM treatment option produces great potential in advanced NSCLC. Here, we hope that more researchers will participate in its follow-up study to further elucidate its long-term therapeutic value and assess possible adverse reactions.

## Author contributions

Conceptualization: Shaoquan Xiong.

Data curation: Ru Li, Hong Ma, Peng Ding, Lilai Hu.

Formal analysis: Jiqiu Qin, Lilai Hu, Shaoquan Xiong.

Investigation: Jiqiu Qin, Ru Li, Hong Ma, Peng Ding, Qin Yang, Lilai Hu, Deliu Wu.

Methodology: Jiqiu Qin, Ru Li.

Supervision: Jiqiu Qin, Ru Li.

Validation: Jiqiu Qin, Peng Ding.

Visualization: Jiqiu Qin, Ru Li, Hong Ma, Qin Yang, Deliu Wu.

Writing – original draft: Jiqiu Qin.

Writing – review & editing: Ru Li, Shaoquan Xiong.
